# Expansion and Marketing of Medicare Advantage to Persons With End-Stage Kidney Disease

**DOI:** 10.1001/jamanetworkopen.2025.16359

**Published:** 2025-06-17

**Authors:** Joan F. Brazier, Amal N. Trivedi, Denise A. Tyler, Renee R. Shield, Emily A. Gadbois

**Affiliations:** 1 Department of Health Services, Policy & Practice, Brown University School of Public Health, Providence, Rhode Island; 2 Scripps Gerontology Center, Miami University, Oxford, Ohio

## Abstract

**Importance:**

The 21st Century Cures Act expanded Medicare Advantage (MA) to include persons with kidney failure. Little is known about the impact of increased MA marketing for persons with end-stage kidney disease (ESKD).

**Objective:**

To examine varied perspectives on the expansion of MA to include persons with ESKD and associated marketing strategies.

**Design, Setting, and Participants:**

This qualitative study of interviews conducted from January 21, 2022, to May 1, 2024, used a modified grounded theory and content analysis approach to analyze transcripts. Interviewees included executives from 8 MA plans, clinical leadership of 5 kidney care management companies, and leadership and staff of 6 dialysis organizations that varied in size (enrollment) from all US Census regions.

**Exposure:**

One-time, semistructured interviews. Interview questions focused on the perceived effects of the expansion of MA to include persons with kidney failure.

**Main Outcomes and Measures:**

Thematic description of participant perspectives on the expansion of MA and associated marketing practices.

**Results:**

A total of 48 interviews with 56 executives and staff from MA plans, kidney care management companies, and dialysis organizations were completed. Analysis found 3 major themes. First, organizations used various outreach approaches to inform persons with kidney failure of their expanded insurance options. Participants recognized that personal choice without undue influence is paramount when older or chronically ill adults are selecting their insurance coverage. Second, MA has potential benefits for persons with ESKD, and organizations expanded partnerships with brokers and/or agencies to provide additional insurance-related resources. Third, the mass marketing approach used to advertise MA was ubiquitous and often reported as deceptive for older or chronically ill adults. Kidney health care professionals noted that constraints on eligibility, restricted health care networks, and limited benefits and services were frequently inadequate for the complex social and medical needs of their patient population.

**Conclusions and Relevance:**

In this qualitative study, findings show that MA marketing to increase enrollment is pervasive and intensifies during open enrollment. Policymakers and industry experts should consider the complexity of insurance selection decisions and monitor accuracy of MA marketing of ESKD-specific benefits.

## Key Points

**Question:**

How do representatives from Medicare Advantage (MA) plans, kidney care management companies, and dialysis organizations perceive the expansion and marketing of MA products for persons with end-stage kidney disease (ESKD)?

**Findings:**

In this qualitative study of interviews with 48 participants from MA plans, kidney care management companies, and dialysis organizations, MA marketing was reported to offer insurance coverage and supplementary benefits that were often inadequate for the complex medical needs of persons with ESKD.

**Meaning:**

These findings suggest that policymakers and industry experts should consider the complexity of insurance selection decisions and monitor the accuracy of MA marketing of ESKD-specific benefits.

## Teaser

This qualitative study assesses changes associated with the 21st Century Cures Act expansion of Medicare Advantage for patients with end-stage kidney disease, particularly the marketing tactics.

## Introduction

Enrollment in Medicare Advantage (MA) plans, private managed care insurers that receive government payments to provide Medicare-covered services, is projected to reach 35.4 million beneficiaries, or more than 50% of the Medicare market, by 2025.^1^ MA plans frequently offer enrollees lower cost-sharing liability in addition to benefits not covered by traditional Medicare (TM) such as dental, vision, and hearing care.^2^ However, unlike TM, MA plans often have restricted provider networks, require prior authorization for certain services, or may deny some health care practices or treatments entirely.^3,4,5,6^ Navigating the complex insurance marketplace is challenging for older or chronically ill adults who must research and select insurance coverage that best meets their medical needs, financial constraints, and health care provider networks.

The rapid growth in MA since 2014 has been accompanied by a sharp rise in MA marketing and sales efforts designed to increase enrollment in MA plans.^7,8^ As MA enrollment has grown, there has been a corresponding increase in consumer complaints that MA marketing is aggressive, confusing, and overwhelming.^8,9,10^ Marketing tactics include the use of celebrity endorsements, images of cards that resemble government-issued Medicare cards, private phone numbers advertised as Medicare hotlines, and suggestions of “missing out” on cash benefits for members,^8^ including higher Social Security payments.^11^ The volume of telemarketing calls, direct mailings, and advertising in the media may be particularly overwhelming during the 9-week Medicare open enrollment period when MA marketing intensifies. One report found that, in 2023, an average of 6 to 9 Medicare-related advertisements per hour was aired on television during the open enrollment period, with 85% of those promoting MA plans.^8^

Prior to the 21st Century Cures Act (hereinafter referred to as the Cures Act), individuals with end-stage kidney disease (ESKD) had more limited insurance options, and were eligible for TM, MA (only if they were enrolled in an MA plan before their diagnosis), or a Special Needs Plan specific to persons with kidney failure.^12,13^ Compounding these insurance limitations for persons with ESKD, in most US states Medigap (ie, Medicare supplemental insurance) may deny coverage or charge higher premiums based on preexisting conditions. In these states, individuals with ESKD enrolled in TM may be unable to obtain a Medigap plan, leaving them vulnerable to significant out-of-pocket costs.^14^ With the implementation of the Cures Act on January 1, 2021, a provision was included enabling persons with ESKD to enroll in MA, an expansion that increased insurance coverage options for those diagnosed with kidney failure.^15,16,17^ Significant growth in MA enrollment of beneficiaries with ESKD has resulted since this policy change went into effect.^18,19^ This qualitative study aimed to understand the changes associated with the MA expansion for persons with ESKD and the marketing tactics targeting this population.

## Methods

This qualitative study presents findings from semistructured interviews with MA plan and kidney care professionals knowledgeable of the Cures Act provision to expand MA eligibility to persons with ESKD. This study followed the Consolidated Criteria for Reporting Qualitative Research (COREQ) guidelines and was determined by the Institutional Review Board of Brown University not to be human participants research. All participants provided oral informed consent to be recorded.

MA plans, kidney care management (KCM) companies, dialysis organization (DO) leadership, and DO site staff were recruited separately and in parallel to each other, using a snowball technique.^20^ Starting with a convenience sample of known contacts, potential participants were identified and recruited by email to participate in semistructured interviews between January 21, 2022, and May 1, 2024. At the completion of each interview, contact information for representatives of other organizations and DO site staff was solicited. In addition, MA plans with high enrollment of beneficiaries with ESKD were purposively identified using the Centers for Medicare & Medicaid Services (CMS) Master Beneficiary Summary File and the CMS MA plan directory and recruited through cold outreach via email. Participants self-identified as knowledgeable about the interview topics.

Interview guides were developed by the research team (J.F.B., R.R.S., and E.A.G.) (eAppendix in Supplement 1), pilot tested with 3 content experts, and edited accordingly for question flow and comprehension. Data from pilot interviews were not included in analyses. To most effectively compare participant responses, the finalized interview guides were designed with baseline questions for all participants and included specifically tailored, open-ended questions with probes targeted for each participant group.

Interviews were conducted by the research team (including J.F.B., D.A.T., R.R.S., and E.A.G.), which consisted of 5 women with 1 to 35 years of experience conducting qualitative research. They included 3 PhD-level faculty, 1 PhD student, and 1 master’s-level research staff member. The research team and the interview participants did not know each other prior to the interview. The research purpose, risks, and benefits were shared during recruitment and at the start of the interview. Interviews were 30 to 60 minutes in length and conducted by telephone or videoconferencing (Zoom [Zoom Communications Inc]), depending on participant preference. Two qualitative research team members (including J.F.B., D.A.T, R.R.S., and E.A.G.) participated in each interview with one leading the interview, while the other took detailed notes, flagged questions for follow-up, and noted emerging themes. Interviews were audio recorded with participant consent. Participants were compensated with a $50 gift card.

### Data Analysis

Interview transcripts were professionally transcribed verbatim, then reviewed and deidentified by 1 research team member (J.F.B.). Transcripts were not shared with participants. Using a modified grounded theory approach,^21^ a preliminary coding scheme was developed based on interview questions (a priori codes) and data emerging from interviews (de novo codes). After testing several transcripts, the team redesigned the coding scheme using a structure, process, and outcomes approach^22^ to facilitate comparison of perspectives across organizations. This coding scheme was adjusted iteratively to add or delete codes and refine coding definitions throughout data collection and analysis. Four researchers (including J.F.B., D.A.T., and E.A.G.) independently coded each transcript, then met weekly to review coding line by line, reconcile coding differences through discussion, and ensure coding rigor. A comprehensive audit trail was kept throughout data collection and analysis, which recorded data collection strategies, identification of emerging primary themes across interviews, and data analysis decisions.^23^ Coded transcripts were entered into the qualitative software package NVivo, version 12 Plus (QSR International). A detailed explanation of participant ID abbreviations is found in eTable in Supplement 1.

At the completion of transcript coding, summary reports were generated in NVivo of all quotations assigned to the same code. The research team then compiled all coding reports considered related to a primary theme identified during data collection and analysis. Each primary theme, and its relevant coding reports, were reanalyzed using a content analysis approach.^24^ During this analysis, data saturation (eg, no additional themes were identified for this subset of data) was ascertained.^25^ For this report, the authors reviewed and analyzed the relevant coding reports for the primary theme of “Patient Experience—Marketing.”

## Results

Semistructured interviews were conducted with administrative and clinical leaders from MA plans, KCM companies, and DOs. MA plans represented all Census regions and collectively enrolled more than 6 million individuals; KCM companies were both regional and national in enrollment; and participating DOs included 2 large national and 4 regional organizations drawn from each Census region. Depending on participant preference, group or individual interviews were conducted. Five group interviews with 2 to 5 leadership participants and 43 interviews with individual participants were completed for a total of 48 interviews with 56 participants. One dialysis organization initially agreed to participate, then later declined due to staff time constraints. Participant demographic information was not formally gathered; however, participants self-reported their position titles and years in the role (eTable in Supplement 1).

Qualitative analysis of interviews with participants from MA plans, KCM companies, and DO leadership and site staff found 3 major themes that reflect perspectives on MA expansion within the insurance marketplace for persons with ESKD. First, various outreach approaches were used to increase awareness of the policy change to expand MA, and organizations acknowledged the importance of personal choice in insurance decision-making. Second, organizations recognized the potential benefits of the MA expansion and expanded their partnerships with brokers and/or agencies to provide additional resources to aid insurance decision-making. Third, participants described MA marketing strategies as designed to increase beneficiary enrollment; however, DO leadership and site staff noted that MA marketing strategies often obscured plan limitations.

### Theme 1: Increasing Awareness of MA Expansion

MA expansion was a major shift in insurance options for persons with ESKD, as it increased their eligibility in the number and type of plans from which they could choose. As a result, MA plans, KCM companies, and DOs shifted their outreach efforts to increase awareness of this policy change. One MA plan executive described working with employer groups “to help message the opportunities that the member has. Whether that’s coming to Medicare Advantage or just leveraging a Medicare contract, you know, a fee-for-service contract” (MA8). A KCM executive noted that their company went “basically on an education tour approaching MA plans that [the policy change] was coming” (KCM5). Similarly, a DO leadership representative reported:

We have a role that we call a benefits navigator, and this is just an insurance educator that is available to help patients answer questions, to inform patients of the different types of Medicare. So not only Medicare Advantage, but original Medicare, the differences between those plans, and the important considerations to take into account when making an insurance decision. (DO2)

DO site staff also participated in informing patients of new insurance options: “I gave [patients] the information and explained to them that this was a new benefit that they might want to be interested in” (SS1).

Organization leadership participants emphasized that in addition to their efforts to increase awareness of the MA expansion, patient choice in the selection of their health insurance was important. Participants from MA plans, DO leadership, and DO site staff were clear about the ethical boundary around influencing a person’s choice of insurance plan. One MA plan executive noted:

There’s just really tight guidelines around how much we can share with [prospective beneficiaries] so that it’s not like we’re guiding them one way or another.…So, we just need to, ethically, make sure that we’re not crossing that line. (MA2)

A DO leadership manager presented this perspective similarly stating, “And that’s really where we keep with the idea that our insurance educators provide information and empower patients to take action or make decisions, but their role in any insurance changes is nonexistent” (DO2). However, messaging around various outreach efforts reflected the complexity between presenting information objectively and unduly influencing a person’s insurance decision. One KCM executive described working with partners on an “outreach campaign” (KCM3), while MA executives and DO leadership described materials for “targeting patients” (MA3) and “identifying…good candidates for MA education” (DO1). One DO site staff social worker described management directives to talk to patients about the expansion of MA as “pressure” to promote MA to their patients (SS1) (Table 1 provides representational quotations).

**Table 1.** Increasing Awareness of MA Expansion

Participant type	Representative quotation (participant ID, role)^a^
**Approaches to increasing awareness of the 21st Century Cures Act policy change to expand MA**	
MA plan	So it’s definitely a space where we know that there’s a portion of the commercial population that have ESRD and so making sure we’re supporting them on the commercial side and helping them in their transition to MA as well. So there are definitely some strategies to support that transition for members and try and give them some more continuity even just in their health care coverage between the commercial and MA. Obviously internally we look to try and figure out which members are the ones that have ESRD and will work with employer groups that have these members on the commercial insurance to help message the opportunities that the member has. Whether that’s coming to MA or just leveraging a Medicare contract, you know, a fee-for-service contract. Obviously, we have a preference for them to transition to MA of course, but so there’s some messaging partnership there with the employer group. (MA8, executive management)
MA plan	As far as communications with members, we’ve had a strategy now for a couple years of just making sure that our members are aware, especially in the realm of the Marketplace line of business, that the members with ESRD are eligible for Medicare. So, we send out a letter to all the members with the diagnosis of ESRD and include in that information on how they can get further details and medicare.gov website, and phone numbers so that the members are aware of that. And we’re in a unique position. From the [MA2] side of things, we couldn’t assist members. There’s some legalities, and we cannot assist members in filling out those applications, but we can make them aware of that. So, that’s what we do. We also have our case manager team outreach to all of our Marketplace members. And if they’re successful in connecting with them, they also educate them about it via telephonic means. (MA2, executive management)
KCM	I would say if you think about all the unique new kidney care companies that started since 2017, they were all directly related to 21st Century Cures Act. That act was signed in, I mean you know this, 2016, but there was a delay in implementation of the embargo against ESRD patients, so that 5-year runway we saw a ton of activity, it was for that reason, people knew that there was going to be a big market. It naturally makes sense for most of our patients with end-stage kidney disease to gravitate toward an MA product. Extra services, enhanced benefit plan design, MOOP, maximum out-of-pocket issues, and so the market took a bet that patients would migrate there. That bet actually is paying off because the uptick of ESRD patients in MA has been great. (KCM3, medical officer)
KCM	I think what happened was…former Senator, majority of [Name], founded the company in 2018 in anticipation of some of these changes. I think [KCM5] prior to me joining went basically on an education tour approaching MA plans that [the policy change] was coming and maybe was also informing some of these executives what was coming in terms of the magnitude. I think there was a lack of awareness around how many patients might choose MA, [and] what that might cost financially for MA plans. And [KCM5] really kind of led the way in explaining that to these providers and created our own business model of going at risk on these patients, because we think we can drive down cost, our in-home model. So I think it was an effort on the companies’ early founders going out and informing, educating. And we had some early adopters who took us up on it in different markets, and the results have been speaking for themselves really in terms of outcomes, driving down costs, increase rates of home dialysis. (KCM5, medical officer)
DO leadership	And so, really, when the 21st Century Cures Act passed, recognizing that it wouldn’t go into effect until 2020, we started the process of developing or staffing up our team and training the team. We have had for a long time at [DO2] insurance educators who are aimed at providing objective and fact-based information to patients so they can navigate their insurance options. And with most of our patients being on Medicare, and most Medicare patients prior to the Cures Act not having really any insurance options or ability to make insurance changes, the amount of education that those patients requested at any given time was fairly light. We recognized that would change with the passage of the 21st Century Cures Act. We have a role that we call a benefits navigator, and this is just an insurance educator that is available to help patients answer questions, to inform patients of the different types of Medicare. So not only Medicare Advantage, but original Medicare, the differences between those plans, and the important considerations to take into account when making an insurance decision. Things like provider networks, cost implications, access to benefits, coordination with other coverage. We grew the size of that team. We trained that team. And simultaneously, we developed a lot of patient-facing materials that we could provide through the mail, through digital education mechanisms to patients. Those materials really closely mirror information that might be on medicare.gov, or might be provided through CMS, or through state health insurance assistance programs aimed at ensuring patients know the different types of Medicare options that are available to them. (DO2, executive leadership)
DO leadership	The one thing that we did have to do though is sort of educate the patients that might’ve fallen in this situation where, because prior to the 21st Century Cures Act, if the patient was diagnosed with ESRD, they couldn’t get an MA plan. So that changed this. So if they were, let’s say for instance, already a Medicare age and they were on Medicare and had a supplement, now they were basically free to choose an MA plan, whereas before they weren’t allowed to do that. So we had to educate the patient that they’re going to get a lot of marketing from plans. They’re going to get told a lot of things that probably aren’t true, especially for their population, because a lot of the MA plans weren’t really familiar with ESRD. So they’re probably going to get told things that may end up being not financially feasible for them. (DO7, executive leadership)
DO site staff	We do have education that does come out from our marketing site when this was taking place, they did send us flyers letting us know that MA was now going to be opening up for ESRD patients. So in terms of that, a lot of the training that I did was with my specific patients. I gave them the information and explained to them that this was a new benefit that they might want to be interested in. And then from there, I partnered with the insurance coordinator at the time to let her know who was interested in possibly talking more about that and possibly getting enrolled in an MA plan as compared to regular Medicare. (SS1, social worker)
**Personal choice: organizations cannot influence the selection of insurance plan**	
MA plan	We show them, at least from the [MA2] perspective, where they can go and look for those opportunities of which ones [insurance plans] would be in network and which ones would not. There’s just really tight guidelines around how much we can share with them, so that it’s not like we’re guiding them one way or another. And then as far as Medicare itself, as you know, these are very expensive members. We don’t ever want to come across that we’re trying to educate the member on this to get them off of our plan and onto another one. So, we just need to, ethically, make sure that we’re not crossing that line, trying to transition numbers away from [MA2], but just trying to make sure that they have the awareness that they can choose a plan that has less out-of-pocket expense for them and more benefits. And then sending them to medicare.gov so that then they have that whole platform of opportunities to look at rather than just trying to promote. (MA2, executive management)
DO leadership	And what [DO1] did was, was pretty substantial. They first of all made sure that the staff in the clinics understood kind of what the rules of engagement were: ie, “Thou shalt not talk to patients about making insurance coverage choices.” That’s a very bright line. We also spent a lot of time educating our medical staff about what you can do and what you can’t do. We went to great lengths to make sure that people understood that when patients were thinking about making this change, you know [Interviewer 1] we felt like it was kind of our obligation to make sure patients knew that all of a sudden they were going to have a choice, but to also make sure they knew that it was an important choice, and we would for example, highlight the fact that MA plans could be a fantastic choice for certain patients and maybe not for others…[DO1] has a huge corporate compliance culture, which is appropriate, and there were tons of educational episodes that tried to ensure that nobody, including the nephrologist, and they obviously don’t work for us, right? but we wanted to make sure the nephrologists, there were very bright lines about what you could and couldn’t say to a patient about….You couldn’t say for example, “Oh, [Participant Name], gosh, I get paid more with Plan A. I think I’m going to tell everybody to go to Plan A.” You know? We bent over backward to try to make sure people know that was one of those go-to-jail [comments] without a get-out-of-jail card. (DO1, executive leadership)
DO leadership	When the 21st Century Cures Act passed, we recognized that we wanted to ensure that patients were able to make well-informed decisions based on objective criteria. And…what I want to point out is that we, as [DO2], as a provider, do not take a stance on what is the best form of insurance for any one patient. We have a really deep belief that insurance decisions are individualized, they’re based on individual patient circumstances. And that’s something that a patient should make with their own interests and objectives in mind. And, we want to ensure that patients have good access to reliable and objective information to make well-informed decisions. (DO2, executive leadership)
DO site staff	Ethically, how to toe that line of “I don’t want to get too involved in their financial and insurance needs.” I just try to give [patients] an umbrella understanding of how insurance works, mostly because that’s my understanding of it, and I don’t want to give out misinformation or steer someone in the wrong direction. (SS6, billing coordinator)
DO site staff	We try not to persuade patients to go this route or that route. I generally say, “Think about what you need.” If you are going to be seeing more than just your primary care, more than just 5 specialists, you know that you’re constantly going to do blood work every month, every 3 months, that’s more than the average person to that just goes for their annual. Sometimes I say having just Medicare in a secondary is not the end-all–be-all. There are other benefits if you looked at a bigger plan, so to say. And sometimes I explain it as a 1-stop shop, you just say, “Well, instead of dealing with 2 separate payers, you’re just dealing with 1. And you can go in and really break down and it will break it down for you to say, ‘Yes, I have to follow with 3 different specialists every 3 months.’” Well, that may pay for itself. But it’s tough because we’re not here to say “You have to choose this one or this is better for you.” It’s more of, “Well, let’s sit down and see what you actually need these services for.” (SS8, social worker)
DO site staff	Because like I said, at the end of the day, it’s the patient’s decision. (SS1, insurance coordinator)
**Outreach and education: personal choice or influence by an organization?**	
MA plan	Again, for our D-SNP business especially, we have a lot of the feet on the street where we have community coordinators in our big markets are [city 1] and [city 2] counties that work directly with the providers to educate them on the benefits that [MA4] offers and what our benefit plans look like. If the providers are seeing patients who perhaps they believe might be dual eligible, they could refer them to us to see if we could enroll them. These folks also participate in a lot of grassroots. It could be like a health fair that’s being done by the county or by a senior citizen center, something like that, that we will participate in and use that as potentially a conduit for folks that might benefit from enrolling in these types of products….I would say the local plans, and maybe not even [MA4], whereas a statewide plan, but the companies that have a strong grassroots footprint tend to be successful in this space. I think that’s really a key to that. And strong relationships with the medical groups, the doctors that are providing the care. Not necessarily….The dialysis centers are really sort of a downstream part of that. They’re already located in those communities. Obviously, they understand where diabetics live, and dialysis centers very much align to those locations. (MA4, executive management)
MA plan	But once someone’s joined from an education standpoint, what we try to do is make sure now that we have several companies that are targeting patients with compromised kidney function, is we’re looking at our data consistently refreshing every couple weeks and sending a list of patients or members that we think might benefit from this type of service. And so that they can call and target the member or target the provider, the PCP, the nephrologist, to make sure that the care is taking place, it needs to take place. And then it’s more of like internal communications that we have. So recognizing that, “Hey, if someone has a compromised GFR, don’t give them Motrin or aspirin if they don’t need it.” And try and tell them what this means and why, and make sure they’re getting the right care. (MA3, executive management)
KCM	We’ve taken a step further with our partners and they’ve actually said also, “Hey, look, these are the patients in your panel, we know from our data these patients haven’t been referred to you yet,” and so we work with them then on an outreach campaign to get patients who are in their local market, based on their claims and lab data, into our practices. So that’s how we do it. Again, we start with our practice, take risk on those panels, and then expand outward from there, not the other way around. (KCM3, medical officer)
DO leadership	We began educating the patients, I believe, very early—probably in some cases too early, because I think a lot of the patients probably forgot about what we told them [laughs], but we started educating them as early as May of 20…what was it? 2019 or 2020? I can’t remember now, 2020, it must have been. And then we did another blitz of educating those patients, both face-to-face and we created a number of different links on our website with additional education, beginning in September of that year. We were able to identify the patients out of our population that we were treating, that would be good candidates for MA education. And so we began educating them face-to-face, the insurance coordinators and the social workers. (DO1, executive leadership)
DO site staff	[MA] must pay better because otherwise, why would [DO1] have shoved it down our throats? We were told to talk to every single patient about switching over…and I was like, “Why are we doing this, if they go to the hospital and need a rehab hospital, they’re not going to get it with these?” They’re like, “No, no, this is a good plan, so you just hmm.”…I didn’t pay that much of attention to it because I didn’t see it as good for the patients, and social work ethics are your loyalty and your advocacy is for the patient, not for the corporation. Our ethics say, you don’t do something that’s bad for a patient and good for your agency. You always, always do what’s best for the patient. (SS1, social worker)
DO site staff	It’s open enrollment time, so it’s all over the TV. Everything is “Switch to this plan, switch to that plan, join this plan, join that plan.” So they’re getting things in the mail, switch to this plan. And they’re getting all types of, I hate to say bribes, but they’re getting all types of, “We will give you this gift card or we’ll do this or you can get this if you switch to this plan.” So that’s one of the difficult things that we have with our patients. And then some patients don’t even know that they’ve been switched to something else. So I think that’s the difficult part of that. (SS7, social worker)

Abbreviations: CMS, Centers for Medicare & Medicaid Services; D-SNP, dual Special Needs Plan; DO, dialysis organization; ESRD, end-stage renal disease; GFR, glomerular filtration rate; KCM, kidney care management; MA, Medicare Advantage; MOOP, maximum out of pocket; PCP, primary care physician; SS, site staff.

^a^ For details on participant characteristics and abbreviations, refer to eTable in Supplement 1.

### Theme 2: Potential MA Benefits and Expanded Partnerships

Participants described the potential benefits of the MA expansion for persons with ESKD. Participants from KCM companies and MA plans called the expansion a “positive change” (KCM3) that focused efforts on improving ESKD care by “trying to reduce hospitalizations, really provide good access to quality primary-level care, access to specialty care, and improve their quality of life and clinical outcomes” (MA3). DO leadership and DO site staff were more cautiously optimistic, expressing that while MA “offers a lot of advantages” (DO3), they also believed that MA should “provide coverage for the patients, that [is] geared more towards what the patient needs” (SS8).

The complexity of insurance options due to the expansion of MA resulted in organizations expanding partnerships with brokers and/or agencies. Participants from MA plans, KCM companies, and DOs described the use of this third party to assist persons with ESKD in assessment of their insurance needs and navigation of insurance options. As 1 DO site staff social worker described the relationship:

Well, today, right now there’s one agency in our area that we deal with a lot. We refer a lot of our patients to them. They’ve just been wonderful through the years helping our patients with coverage. So this year for the first time, we actually have that particular agency coming into the centers and helping people look at coverage, looking at their medicine. And so that’s been helpful. (SS6)

Additional representative quotations are provided in Table 2.

**Table 2.** Potential MA Benefits and Expanded Partnerships

Participant type	Representative quotation (participant ID, role)^a^
**Potential MA benefits for persons with ESRD**	
MA plan	Generally, we were supportive of the [policy] change because we look at what we’ve been doing in MA with non-ESRD patients and our focus just being around trying to reduce hospitalizations, really provide good access to quality, primary level care, access to specialty care, and improve their quality of life and clinical outcomes. The thought is, can you do this with ESRD? That’s why they’re making the push and it makes sense to do. And then the more you learn about the population, the ESRD members not having a maximum out-of-pocket cost, you can’t survive. If we can get them MA and have a max out-of-pocket cost for them, and actually improve them, it makes sense. (MA3, executive management)
MA plan	I mean, this is my own bias, because coming from a[n] MA plan and a medical group that was supporting with more HMO type strategies, like you heard me say we were providing better care at half the cost of a straight Medicare patient. And so in my mind, it was almost like, “Why didn’t they do that sooner? What was the drawback to not encouraging patients to be MA?” because they need so much coordination of care. There’s so many opportunities for poor coordination in straight Medicare and outcomes being affected, leading to higher cost, or just other strategies just leading to higher cost. And so if you have a system that really does focus on coordination and making sure that whatever their needs are getting addressed timely, that’s the difference that you make for ESRD patients. (MA6, executive management)
KCM	So, I think MA allows the provision of services that wouldn’t necessarily be allowed in fee for service, per se, that you can bill. And so in my opinion, it’s a win for most patients. I mean, I can’t generalize. There’s some people that may prefer fee for service for other reasons, but I think the ability to provide other types of services, especially around behavioral health and social determinants and things like that, is a pretty big win. And so the way I see it is that any value-based arrangement will be good, will be as good as the opportunity and the ability to provide all these ancillary services that patients need to be able to deliver them appropriately. And so that’s the biggest difference I see is really all these other additional services that a patient might be now eligible for. (KCM2, medical officer)
KCM	I think this is a positive change. I’m a big proponent. If you would’ve asked people from payer organizations 5 years ago, they were really scared because they were like, “We’re going to get the sickest group of people now on our rolls and we don’t have any history on, we can’t adjust for, you can’t risk adjust for, we don’t know.” So they were scared to death. From a provider and patient experience perspective, this is such a good thing. I keep saying this again and again and again, but it’s really important to underscore, having access to limited maximum out of pockets, I mean most people who are 65, 70, unless you’ve done really well in life, have some limited income you’re living on, it may be a lot, but that really is helpful. Then just having a coordinated one-stop shop approach with MAPD, so Part D drug plan integrated with all of your medical needs, plus all the extra benefit enhancements that come with MA, again remember, like I said, transportation is a big one, most of my patients use that benefit in some shape, form, or fashion because a lot of them are counting on family members driving them to appointments. Sometimes people can’t do that, and so to be able to have that benefit to go to a doctor’s appointment or dialysis is really key. You’d be surprised that a lot of people using their payer benefits around food insecurity, so food vouchers, food cards, that stuff is really important. I think this is a positive change, especially for this really vulnerable population that has a ton of social and structural determinant issues that are driving their health care outcomes, people with end-stage kidney disease or advanced kidney disease. I think this is mostly a positive thing. I’m a big proponent. Like I said, MA plans would probably disagree with that, but I think it’s a step in the right direction. (KCM3, medical officer)
DO leadership	I think that [MA] on paper offers a lot of advantages. Certainly I, like everybody else, see the commercials on television for all the wonderful things that these MA plans offer, and I worry that some of our patients, particularly the less sophisticated ones, may have a difficult time kind of sorting through the ads on television vs [their medical] reality. (DO3, executive leadership)
DO leadership	So this is a very vulnerable population in particular. I think that’s why it’s so important to look at this. These are some of the folks who are the most socioeconomically disadvantaged. They have the greatest burden of chronic illness. It substantially impacts the BIPOC community to a greater degree than others. If you think about people and backgrounds, people on dialysis, if you think about social determinants of health, there are a lot of things to consider. So if MA plans can provide additional services to a fragile and vulnerable community, great. But I do see the MA plans scrambling for people, you know, they’re cherry picking and lemon dropping, that’s something else to kind of pay attention to. But I do worry because a lot of times these things always come down to a dollar as opposed to a patient outcome and quality. (DO4, executive leadership)
DO site staff	I just think that ESRD is such a niche illness and I think that there’s a lot that goes along with it and that sometimes people have to change medications because it’s not working properly or not that the dialysis isn’t working, but do you know what I mean? That they have different needs along with their other comorbid issues. And I think that if [MA plans are] trying to help the patients or at least provide coverage for the patients, that it should be geared more toward what the patient’s needs are vs some of the other things that they’re doing. (SS8, social worker 1)
DO site staff	Well, I think that they should offer benefits that would, I guess, complement their disease and possible options. Do you know what I mean? Instead of, I think that they should be gearing it more toward accommodating the costs of having ESRD vs a lot of the fluff things that they do offer. [Like] gym membership. (SS8,social worker 2)
**Expanded partnerships with brokers and/or agencies**	
MA plan	I think brokers would’ve been aware that they could sign up. That varies from MA plan to MA plan, whether they use an internal sales force. The market in [State] is very broker driven. I think probably plans in [State], 90% of MA sales probably come through brokers. So brokers would’ve been made aware of that. Our existing membership was certainly aware of the programs that we had set up and were already engaged in those. (MA4, executive management)
MA plan	Internally we look to try and figure out which members are the ones that have ESRD and will work with employer groups that have these members on the commercial insurance to help message the opportunities that the member has. Whether that’s coming to MA or just leveraging a Medicare contract, you know, a fee-for-service contract. Obviously, we have a preference for them to transition to MA…but so there’s some messaging partnership there with the employer group. (MA8, executive management)
KCM	But I think there’s a lot of moving parts here…patients can choose MA during open enrollment, MA plans have brokers et cetera that are driving patients for choosing them, perhaps I think some of that has come to realization that people are choosing MA particularly those with ESRD or potentially having ESRD. (KCM5, medical officer)
DO leadership	So we partnered with an organization, it was called [company name], and what we would have our insurance coordinators do is say, “Hey, you’ve never had this opportunity before, but you have an opportunity to choose to step into an MA plan, and you really should talk to a licensed insurance broker. We’ve worked with one that we are basically providing a list to all of your medications and all of your doctors. And we know if you’re on a transplant list and we’re telling them which transplant center you’re on.” So we tried to say…we obviously weren’t steering people for that insurance broker, but by kind of collaborating with one, we had basically a way to shift important data so that a patient wouldn’t show up in front of their insurance broker and not have their medication list for example, or forget the name of their cardiologist, or where they were listed. So we did a lot of that. (DO1, executive leadership)
DO leadership	At the beginning, we didn’t [engage with patients]. This year, we’re doing something very different. I have engaged a benefits consultant, like certified Medicare, MA benefits consultant and we’re opening our doors to them. We have a couple of different days at each of our centers where they’re coming in. We’ve signed a nondisclosure with them. The patient, they can raise their hand and say, “I want to meet.” or not. Schedule times with them. We’ve provided our patients with a blanket agreement where we’re allowed to share information with the benefits consultant if they so allow, and it includes demographics, medication list, problem list, hospitalization, history, so that they can get an idea of forecasting what their out-of-pocket spend is going to be. (DO6, executive leadership)
DO site staff	So these third parties, so these are agents. These are companies, too. They are, let’s say broker agencies, insurance broker agencies, and local agents, independent agents as well. The company itself, they’re doing the national type, the ones with the broker agencies. Us, insurance coordinators, we partner with our local agents or brokers, you know, smaller, independent agencies because we know them and we can talk with them if we had to just explain, or we may have one specific patient who needs that one on one. Then I will refer them to them, because that’s what they need. If it’s a more of a person who can do their own, then we can refer them to the larger source, which is maybe a bigger, like website or something easier. They already know what they want. We give them the information. They’re like, “Okay, I’m ready.” They can work. I can refer them to them. No problem. (SS1, insurance coordinator)
DO site staff	Well, today, right now there’s one agency in our area that we deal with a lot. We refer a lot of our patients to them. They’ve just been wonderful through the years helping our patients with coverage. So this year for the first time, we actually have that particular agency coming into the centers and helping people look at coverage, looking at their medicine. And so that’s been helpful. And we do have patients that are really taking advantage of that. (SS6, specialist)

Abbreviations: BIPOC, Black, Indigenous, and people of color; DO, dialysis organization; ESRD, end-stage renal disease; HMO, health maintenance organization; KCM, kidney care management; MA, Medicare Advantage; MAPD, MA Part D; SS, site staff.

^a^ For details on participant characteristics and abbreviations, refer to eTable in Supplement 1.

### Theme 3: MA Marketing Practices and Challenges

To increase beneficiary enrollment, MA plans market their products to the general population eligible for Medicare. Participants from MA and KCM companies were careful when discussing marketing strategies and noted that marketing was not “tailored specific[ally] to an ESRD [end-stage renal disease] member because the volume, the percentage, is very small and you’re not really supposed to target [a] certain member vs the other” (MA3). A DO site staff social worker described the global approach to marketing MA as:

I don’t think that health care companies, the private health care companies specifically, target ESRD populations, because I still think that they don’t probably make money on these patients. But the marketing in general that’s out there is overwhelming, and it’s presented that “you’re missing out on something if you don’t have Medicare Advantage.” (SS1)

MA marketing to enroll new beneficiaries also intensifies during open enrollment periods. As one DO leadership manager reflected:

I would say that one of the challenges…for the patients is the bombardment of the information during these open enrollment periods.…It’s on television, you hear it on the radio. I mean, it’s everywhere. And I think that causes a lot of confusion. (DO1)

DO leadership and DO site staff further reported how MA marketing promised expanded benefits and services but did not specify eligibility requirements or the limitations on those benefits and services—a tactic they called “deceiving” (DO1). Network restrictions on transplantation services, transportation, specific formularies for medicine, and access to specialists and providers have large impacts on access to necessary medical care for those with kidney failure. As a DO leadership manager described, “[Patients with ESRD were] finding out that [their] transplant center was no longer willing to talk to [them] because [they] had changed to some plan that they weren’t welcome in” (DO1). This was further observed by a DO site staff social worker:

We have had cases where there are consumers who are choosing these plans or these plans are presented to them because of the advantages or benefits that it offers, such as over [the] counter card[s], the things in the pharmacy and the supermarket, things of that nature. And it’s appealing, I get it, but oftentimes [patients] don’t realize that yes, it does cover dialysis, but it’s not in network with [us] or it’s not in network with their ophthalmologists or their dietician. (SS3)

Persons with ESKD “…are getting lost in the mix,” another DO site staff billing coordinator reflected, “and I think that’s what bothers me the most” (SS6). Table 3 shows more representative quotations.

**Table 3.** MA Marketing Practices and Challenges

Participant type	Representative quotation (participant ID, role)^a^
**MA plan marketing is global and geared toward all who are eligible**	
MA plan	I know our plan hasn’t put out signage or marketing material to say, “Have kidney disease? Come over here.” And I’m not saying we don’t do that for any other disease conditions. You don’t say, “Diabetes? We’re your plan.” But I haven’t seen that. (MA7, executive management)
MA plan	So there’s education for choosing a plan and then there’s education once you’re in the [MA3] plan. Now for choosing a plan, I think we work with a lot of agents that go and help try and choose a plan. And I think we try and mirror those kind of decisions that help you. But I don’t think there’s anything tailored specific to an ESRD member because the volume, the percentage is very small and you’re not really supposed to target certain member vs the other. Like member adverse member selection. (MA3, executive management)
KCM	I’m going to say [marketing strategy] probably varies by plan. I don’t know how much advertising they do, per se, to get folks to sign up. I’m not sure. (KCM2, medical officer)
KCM	I think how they [ESRD patients] make that decision is probably almost at random. I have never had a patient share with me that “I carefully went down all these different metrics of these different plans and I made this decision.” I’ve never once had a conversation and I am full-time clinical in addition to being half-time at [KCM4]. It has never come up. And so I suspect either their adult children are signing them up for it or they worked with one of these Medicare advisory people. Or they got a flyer in the mail or some nice person on the phone called them but I don’t think that there’s been…yeah. That is something that has never, ever come up. (KCM4, medical officer)
DO leadership	I would say that one of the challenges, and I don’t think that this is a challenge necessarily for us, but I think it is for the patients, is the bombardment of the information during these open enrollment periods, because we’re providing them with information, it’s on television, you hear it on the radio. I mean, it’s everywhere. And I think that causes a lot of confusion. I think that there’s another side of that as well. I think that a lot of these companies that are providing this advertising, brokers in particular, are very good at what they do. They do not focus on one plan or one type of plan. I think they do provide really good education and analysis of what they currently have vs what’s available to them. (DO1, executive leadership)
DO leadership	The MA plans are….You have all these people on TV selling it, and then you have the hospital systems with their own plans pushing it [MA] as well. (DO6, executive leadership)
DO leadership	I can’t imagine being a patient and walking into a grocery store and there’s a pop-up table with someone selling insurance and just saying, “Oh yeah, for sure I’m doing that.” And so we know some patients fall into that, or they get cold-called from brokers or they see the TV commercials from brokers and it promises so much. I know for me I’ve sat on the couch and watched those commercials for years and just thought, “So deceiving!” Because this isn’t going to work for so many people. (DO1, executive leadership)
DO site staff	And so in the marketing, I may be moving ahead of y’all’s questions, but the marketing for these MA plans were unrelenting. Even to the point I’m not even sure that the marketers even, probably…I don’t know. I don’t think that health care companies, the private health care companies specifically target ESRD populations, because I still think that they don’t probably make money on these patients. But the marketing in general that’s out there is overwhelming, and it’s presented that “You’re missing out on something if you don’t have Medicare Advantage.” These patients would call without any other [insurance] knowledge, would [talk about] some celebrity on a CNN commercial [who] says that you would benefit, you’re missing out on a benefit if you don’t have that MA stuff. (SS1, social worker)
DO site staff	I think the worst aspect of the system, from where I’m sitting, is the idea that these insurance companies will contact the patients directly, and promise them the sun, the moon, and the stars, only to turn around and the patient finds out, “Yeah, no.” Because they’re not looking at the patient’s best interest. They [patients] don’t understand, or they’re not, I mean, it’s just, like I said, there’s a lot of information being pushed at them at once.…I think the idea that insurance companies contact the patients directly…should be eliminated. I mean, your brokers are a little more apt to sit down with a patient and their family, hopefully, especially if they’re older, and look at the whole picture. (SS1, patient account manager)
DO site staff	Most of my patients who say they enroll saw an ad on TV or on the subway, or had a neighbor who enrolled. But again, they’re not dealing with necessarily the same comorbidities, and the frequency of appointments. It’s just, what works for your neighbor, unfortunately does not always work for the patient. Yeah, that’s typically how they [ESRD patients] know about it, is [through] a family member, a friend, an ad, and they are enticed by what the services say that [the MA plan] can provide. And I think that [the MA plan] could work for a lot of patients, I just haven’t seen it really work well for my dialysis patients. (SS3, social worker)
**Dialysis providers and site staff note that the global nature of MA marketing highlights benefits for all and obscures plan limitations**	
DO leadership	I think what we’ve kind of found the last year or so is that even though there’s a transportation benefit, it depends on the [MA] plan and it might be limited.…Like that’s the bigger issue that we’ve heard more of this last year is “That’s great there’s transportation, but I might get 10 rides a year,” which for in-center patients they’re at the clinic maybe 13 times [a month]. So that’s already maxed out within a month. (DO1, executive leadership)
DO leadership	So, we had to educate the patient[s] that they’re going to get a lot of marketing from plans [and] they’re probably going to get told things that may end up being not financially feasible for them.…So, for instance, if a patient had traditional Medicare and had a supplement, and that supplement was one of those plans that I know they might be grandfathered now. So, there was plans out there that paid the coinsurance and things like that, and maybe it was $250 a month. Well, switching to an MA plan might cost them more money even though at some point they’ll meet the out-of-pocket maximum. So, most of our patients are hitting the out-of-pocket maximum every year: they’re going to go to the hospital, the dialysis itself. If they pay 20% of whatever the payment rate is, they’re going to hit the out-of-pocket max likely somewhere in the middle of the year. So even with that though, if you’re paying $200 to $250 a month for 12 months, that’s only $2500 to $3000 a year where the out-of-pocket max might be $6500. So, you may be worse off financially, even though you’re promised vision and dental and all this other stuff that really is, in the grand scheme, is insignificant to a dialysis patient. However, [dialysis patients] do really need to have a good dental plan because they need that for transplant. If they don’t have their teeth checked and things like that, they might not get on a transplant list. (DO7, executive leadership)
DO leadership	I guess the thing that surprised us most, and what we’re trying to learn from is the patients that switched back pretty quickly. And so we’ve been carefully trying to get a sense of, “Was it the fact that you couldn’t get the medications you wanted? Was it the fact you couldn’t go see a specific provider?”…That one has cropped up. Finding out that your transplant center was no longer willing to talk to you because you had changed to some plan that they weren’t welcome in. And then occasionally, patients would find their nephrologist is not in network with the plan they switched into. And that really created some angst. (DO1, executive leadership)
DO site staff	We have had cases where there are consumers who are choosing these plans or these plans are presented to them because of the advantages or benefits that it offers, such as over counter card, the things in the pharmacy and the supermarket, things of that nature. And it’s appealing, I get it, but oftentimes they don’t realize that yes, it does cover dialysis, but it’s not in network with [DO3] or it’s not in network with their ophthalmologists or their dietician. So my job is pretty much try to educate them on that and also kind of do the numbers with them and show them, this may not work, but maybe this plan will, and this is how much you’re saving per year if you choose this plan over that plan. (SS3, social worker)
DO site staff	The patients are getting lost in the mix, and I think that’s what bothers me the most. They’re being looked at, I think, by the insurance companies, as a financial commodity or a financial liability. And I don’t see where the patients are benefiting from my perspective. (SS6, specialist)
DO site staff	I want to say that I still think it sounds too good to be true.…They’re disappointed. They change [to an MA plan] and they’re glad about the decrease in the Part B premium, but then they come into an issue if they need some assistance with transportation, or if there’s an issue of a particular phosphate binder that they need that they can’t afford. And it’s difficult to get those 2 things. And then when they try the vision and the dental, it’s like $100 for each service, which doesn’t really do a whole lot, when you’ve got a whole lot that needs to be done. Some of our patients need cataract surgery, need glasses, they need drops. You know? It’s just ongoing stuff that they need, and $100 extra doesn’t really go a long way. Dental work, $100 doesn’t really help a whole lot. (SS1, social worker)

Abbreviations: DO, dialysis organization; ESRD, end-stage renal disease; KCM, kidney care management; MA, Medicare Advantage; SS, site staff.

^a^ For details on participant characteristics and abbreviations, refer to eTable in Supplement 1.

## Discussion

Findings from this qualitative study reflect perspectives from MA plan executives, KCM clinical leadership, and DO leadership and site staff on the Cures Act policy change to expand MA to persons with ESKD. The expansion of MA increased insurance options for persons with ESKD, which, in turn, expanded MA outreach efforts to increase enrollment of beneficiaries with ESKD. In response, DO processes for providing resources to patients about insurance also grew to address the increased complexity of insurance options. According to interview participants, the shift in MA outreach to enroll persons with ESKD frequently included the use of marketing practices they considered misleading. Although MA plans may provide extra benefits, such as gym memberships, transportation, and monetary gift cards, these same plans may have restricted networks or other barriers for critical services, such as kidney transplantation, for persons with ESKD. Three important implications emerged from this study’s findings.

First, as participants reported, MA marketing was ubiquitous and frequently overwhelming, especially during open enrollment periods. Research has shown that the process for choosing insurance, whether TM or MA, is challenging for eligible beneficiaries, especially when resources for information on insurance options are difficult to find.^8^ MA products are directly marketed to older adults by agencies, brokers, private insurance companies, mailings, and various media platforms. In contrast, and unlike MA, neither the federal government nor any other organization directly markets TM.^7^ This unequal advertising is particularly pronounced during the annual Medicare open enrollment period. This is noteworthy as, during open enrollment, MA marketing significantly increases while the limited marketing of TM information remains unchanged—a disparity that makes navigating the insurance marketplace additionally difficult for older adults. Addressing the lack of easily available resources for information on insurance options, as well as reducing the skewed advertising differential toward MA, is critically needed.

Second, our findings showed that MA and KCM executives, DO leadership, and DO site staff all reported on the ethical boundary to not influence personal choice in insurance selection. This clear line blurs, however, over the definitions of marketing, education, and communication about MA products. Interview participants used this terminology differently. For example, none of our participants used the term *marketing*. Instead, MA plan executives referred to their outreach efforts as *communications* or *messaging*. KCM executives used *outreach*, and DO leadership and site staff *educated* patients and developed *educational* materials. With the sharp rise in MA enrollment since 2014, there has been a corresponding growth in beneficiary complaints of aggressive and misleading MA marketing, and violations of federal rules around MA marketing and advertising.^8,10,16,26^ In response, CMS issued new rules that took effect in 2022 for marketing, education, and communications around MA products, policies for brokers selling MA plans, and guidelines for clinicians discussing insurance with their patients.^7,27^ Despite the implementation of new rules around MA marketing, beneficiary issues and complaints with MA marketing have persisted.^3^ As policymakers consider further refining rules around MA marketing practices, they should consider clearly defining *marketing*, *communication*, *outreach*, and *education* to reduce deceptive marketing practices.

Third, participants from DOs considered the global approach of MA marketing as misleading for persons with ESKD, a particularly vulnerable population due to their unique medical needs. While participants from all organizations noted the potential benefits of MA plans for persons with ESKD, DO leadership and site staff reported that MA plan eligibility requirements, network restrictions, and benefit limitations were not clearly specified in MA marketing literature, in television and radio advertisements, or by brokers selling MA products. They also observed that despite the expanded insurance options for those with ESKD, MA plan benefits were often inadequate for their patients and noted that addressing underlying comorbidities, medication changes, shifts in dialysis modalities during the course of disease progression, access to transplantation, and availability of palliative care services are critically important for ESKD medical care. Policymakers and industry experts should monitor the availability of ESKD-specific benefits such as coverage for pharmaceutical formularies, expanded transportation for in-center dialysis, dental care in preparation for kidney transplant, support services for in-home dialysis, and the adequacy of networks for specialty nephrology, dialysis, and transplantation services.^28^ Last, in contrast to participants from MA plans and KCM companies who stated that MA will reduce out-of-pocket costs for members with ESKD, DOs reported that their patients were switching to MA to receive monetary gift cards and supplemental benefits, not for the lower copayments that MA might provide. This finding suggests that while research on actual out-of-pocket costs for persons with ESKD is preliminary,^29,30^ MA marketing may be obscuring important financial information that should be considered when selecting health insurance.

### Limitations

This study has limitations. First, although we included a large study sample for qualitative analyses, our findings may not be generalizable to all MA plans, KCM companies, and DOs throughout the country. It is possible that those who opted not to participate in this study may have alternative perspectives and approached the policy change to expand MA differently from the viewpoints presented herein. Second, although we present varied perspectives from clinical, administrative, and health system leaders about the association of the Cures Act policy changes with expansion of MA to beneficiaries with ESKD, we did not interview persons with ESKD and thus are unable to present their points of view. Future research is needed to understand the long-term effects of this MA expansion on this population. Third, various policy changes and payment models, such as the ESRD Treatment Choices Model and Kidney Care Choices Model, occurred concurrent with and after the Cures Act in 2021. Research is needed to explore these competing payment models within the context of MA expansion, as these payment models may have influenced marketing, advertisements, and enrollment into MA. Despite these limitations, our sample presents standpoints from leadership of MA plans that varied in size and geographic location, perspectives from personnel of national and regional DOs, and stances from executive-level to front-line staff.

## Conclusions

In this qualitative study of clinical, administrative, and health care leaders in kidney care, findings showed that for persons with ESKD, researching and selecting insurance coverage that best suits their complex medical needs, financial constraints, and health care provider networks was challenging. This study also highlighted potentially misleading marketing tactics targeting this medically vulnerable population and the engagement of brokers and/or agencies by DOs to provide resources to patients navigating the insurance marketplace. Further research is needed to better understand the long-term impacts of MA on health outcomes for persons with ESKD and the effects of limited networks, preauthorization processes, and inadequacy of supplemental benefits for dialysis and transplantation services. Policymakers should consider the significant role marketing plays in insurance selection decisions and take steps to curtail aggressive and misleading marketing tactics.

## Notes

Supplement 1. **eAppendix.** Interview Guides **eTable.** Organization Characteristics and Participant Experience

Supplement 2.
Data Sharing Statement

## References

[1] Ramsey C, Jacobson G, Findlay S, Cicchiello A. Medicare Advantage: a policy primer, 2024 update. *Explainer*. Commonwealth Fund. January 31, 2024. Accessed March 20, 2025. https://www.commonwealthfund.org/publications/explainer/2024/jan/medicare-advantage-policy-primer

[2] MedPAC Report to the Congress: outpatient dialysis services. March 2024. Accessed March 20, 2025. https://www.medpac.gov/wp-content/uploads/2024/03/Mar24_Ch5_MedPAC_Report_To_Congress_SEC.pdf

[3] Aaron DG, Cohen IG, Adashi EY. Medicare Advantage under fire: public criticism and implications. J Gen Intern Med. 2024;39(14):2849-2852. doi:10.1007/s11606-024-08876-7 38926321 PMC11534905

[4] Feyman Y, Figueroa J, Garrido M, Jacobson G, Adelberg M, Frakt A. Restrictiveness of Medicare Advantage provider networks across physician specialties. Health Serv Res. 2024;59(4):e14308. doi:10.1111/1475-6773.14308 38594081 PMC11250170

[5] Meyers DJ, Belanger E, Joyce N, McHugh J, Rahman M, Mor V. Analysis of drivers of disenrollment and plan switching among Medicare Advantage beneficiaries. JAMA Intern Med. 2019;179(4):524-532. doi:10.1001/jamainternmed.2018.7639 30801625 PMC6450306

[6] Meyers DJ, Rahman M, Trivedi AN. Narrow primary care networks in Medicare Advantage. J Gen Intern Med. 2022;37(2):488-491. doi:10.1007/s11606-020-06534-2 33469747 PMC8810971

[7] Findlay S, Jacobson G, Leonard F. The role of marketing in Medicare beneficiaries’ coverage choices. *Explainer*. Commonwealth Fund. January 5, 2023. Accessed March 20, 2025. https://www.commonwealthfund.org/publications/explainer/2023/jan/role-marketing-medicare-beneficiaries-coverage-choices

[8] Biniek JF, Cottrill A, Sroczynski N, . How health insurers and brokers are marketing Medicare. Kaiser Family Fund. September 20, 2023. Accessed March 20, 2025. https://www.kff.org/medicare/report/how-health-insurers-and-brokers-are-marketing-medicare/

[9] Freed M, Cottrill A, Biniek JF, Neuman T. What do people with Medicare think about the role of marketing, shopping for Medicare options, and their coverage? Kaiser Family Fund. September 20, 2023. Accessed March 20, 2025. https://www.kff.org/medicare/report/what-do-people-with-medicare-think-about-the-role-of-marketing-shopping-for-medicare-options-and-their-coverage/

[10] Jacobson G, Leonard F, Sciupac E, Fisch-Friedman M, Rapoport R. The private plan pitch: seniors’ experiences with Medicare marketing and advertising. Commonwealth Fund. September 12, 2023. Accessed March 20, 2025. https://www.commonwealthfund.org/publications/issue-briefs/2023/sep/private-plan-pitch-seniors-experiences-medicare-marketing-advertising

[11] The United States Committee on Finance. Deceptive marketing practices flourish in Medicare Advantage. November 2022. Accessed March 20, 2025. https://www.finance.senate.gov/imo/media/doc/Deceptive%20Marketing%20Practices%20Flourish%20in%20Medicare%20Advantage.pdf

[12] Kirchhoff SM. Medicare coverage of end-stage renal disease (ESRD). Congressional Research Service. August 16, 2018. Accessed March 20, 2025. https://sgp.fas.org/crs/misc/R45290.pdf

[13] Morgan PC, Kirchhoff SM. Medicare Advantage (MA) coverage of end stage renal disease (ESRD) and network requirement changes. Congressional Research Service. January 1, 2021. Accessed March 20, 2025. https://www.congress.gov/crs-product/R46655

[14] Meyers DJ, Trivedi AN, Mor V. Limited Medigap consumer protections are associated with higher reenrollment in Medicare Advantage plans. Health Aff (Millwood). 2019;38(5):782-787. doi:10.1377/hlthaff.2018.05428 31059373 PMC6541000

[15] 114th Congress. 21st Century Cures Act. Public Law 114–255 December 13, 2016. 130 Stat 1033-1344. Accessed December 1, 2024. https://www.congress.gov/114/plaws/publ255/PLAW-114publ255.pdf

[16] Centers for Medicare & Medicaid Services. Medicare and Medicaid programs; contract year 2021 and 2022 policy and technical changes to the Medicare Advantage program, Medicare prescription drug benefit program, Medicaid program, Medicare cost plan program, and programs of all-inclusive care for the elderly. *Fed Regist*. 2021;86(11):5864-6135.

[17] Nguyen KH, Oh EG, Meyers DJ, Kim D, Mehrotra R, Trivedi AN. Medicare Advantage enrollment among beneficiaries with end-stage renal disease in the first year of the 21st Century Cures Act. JAMA. 2023;329(10):810-818. doi:10.1001/jama.2023.1426 36917063 PMC10015314

[18] Nguyen KH, Oh EG, Meyers DJ, . Medicare Advantage enrollment following the 21st Century Cures Act in adults with end-stage renal disease. JAMA Netw Open. 2024;7(9):e2432772. doi:10.1001/jamanetworkopen.2024.32772 39264629 PMC11393715

[19] Morgan PC, Kirchhoff SM. Medicare Advantage (MA) coverage of end stage renal disease (ESRD) and network requirement changes. CRS Report R46655. January 11, 2021. Accessed March 20, 2025. https://crsreports.congress.gov/product/pdf/R/R46655

[20] Parker C, Scott S, Geddes A. Snowball sampling. In: Atkinson P, Delamont S, Cernat A, Sakshaug JW, Williams RA, eds. SAGE Research Methods Foundations. Sage Publications Ltd; 2019.

[21] Strauss A, Corbin J. Grounded theory methodology: an overview. In: Denzin NK, Lincoln YS, eds. Handbook of Qualitative Research. Sage Publications; 1994:273-285.

[22] Donabedian A. Evaluating the quality of medical care. 1966. Milbank Q. 2005;83(4):691-729. doi:10.1111/j.1468-0009.2005.00397.x 16279964 PMC2690293

[23] Ritchie J, Lewis J. Qualitative Research Practices: A Guide for Social Science Students and Researchers. Sage Publications; 2012.

[24] Curry LA, Shield RR, Wetle TT, eds. Improving Aging and Public Health Research: Qualitative and Mixed Methods. American Public Health Association and Gerontological Society of America; 2006.

[25] Saunders B, Sim J, Kingstone T, . Saturation in qualitative research: exploring its conceptualization and operationalization. Qual Quant. 2018;52(4):1893-1907. doi:10.1007/s11135-017-0574-8 29937585 PMC5993836

[26] Novick B. Medicare Advantage and deceptive marketing. Center for Economic Policy Research. November 7, 2023. Accessed March 20, 2025. https://cepr.net/report/medicare-advantage-and-deceptive-marketing/

[27] Jaffe S. Uncle Sam wants you … to help stop insurers’ bogus Medicare Advantage sales tactics. Kaiser Family Foundation Health News. November 30, 2023. Accessed March 20, 2025. https://kffhealthnews.org/news/article/medicare-advantage-deceptive-sales-tactics-federal-crackdown/?utm_campaign=KHN%3A%20Topic-based&utm_medium=email&_hsmi=286559211&_hsenc=p2ANqtz--_8OdwGMzt0HPA9mYPjQfF5-

[28] Oh EG, Meyers DJ, Nguyen KH, Trivedi AN. Narrow dialysis networks in Medicare Advantage: exposure by race, ethnicity, and dual eligibility. Health Aff (Millwood). 2023;42(2):252-260. doi:10.1377/hlthaff.2022.01044 36745840 PMC10837791

[29] Freed M, Biniek JF, Damico A, Neuman T. Medicare Advantage in 2024: premiums, out-of-pocket limits, supplemental benefits, and prior authorization. Kaiser Family Fund. August 8, 2024. Accessed March 20, 2025. https://www.kff.org/medicare/issue-brief/medicare-advantage-in-2024-premiums-out-of-pocket-limits-supplemental-benefits-and-prior-authorization/

[30] Park S, Meyers DJ, Trivedi AN. Association of Medicare Advantage enrollment with financial burden of care: a retrospective cohort study. Ann Intern Med. 2024;177(7):882-891. doi:10.7326/M23-2480 38914004

