# Supplementary material for: Expansion and Marketing of Medicare Advantage to Persons With End-Stage Kidney Disease
Source: JAMA Netw Open. 2025 Jun 17;8(6):e2516359. doi: 10.1001/jamanetworkopen.2025.16359 (PMC12175025; doi:10.1001/jamanetworkopen.2025.16359)
Supplement: Supplement 1. — eAppendix. Interview Guides eTable. Organization Characteristics and Participant Experience [file jamanetwopen-e2516359-s001.pdf]

Supplementary Online Content

Brazier JF, Trivedi AN, Tyler DA, Shield RR, Gadbois EA. Expansion and marketing of Medicare Advantage to persons with end-stage renal disease. *JAMA Netw Open.* 2025;8(6):e2516359. doi:10.1001/jamanetworkopen.2025.16359

eAppendix. Interview Guides

eTable. Organization Characteristics and Participant Experience

This supplementary material has been provided by the authors to give readers additional information about their work.

© 2025 Brazier JF et al. *JAMA Network Open*.

eAppendix. Interview Guides

Medicare Advantage Plan Interview Guide:

Introductory and consent to audio record language

Background:

- What is your role at this MA plan? *Probes: How long have you been in this role? How did you first learn of the change in eligibility for individuals with end stage renal disease or ESRD?*

Preparation:

- What did your plan do to prepare for the change? *Probe*: *Did you give any feedback to CMS about the eligibility change?*
- How did you engage with members regarding the expansion in eligibility?
- How did you work with dialysis providers to prepare for the expansion in eligibility? *Probe: What about the resulting increase in possible patients?*
- How did you evaluate or modify the network of dialysis centers?

Care Processes:

- How does your plan try to improve care for persons with ESRD?
- What is a strategy that your plan has implemented to improve care for people with ESRD? *Probes: How is it going? How do you know if it's working?*
- We're interested in how your plan addresses in-home dialysis and transplantation. *Probes: What role does MA have in the selection of in-home versus in-center dialysis? We've heard about the benefits of home-hemodialysis - what do you think? What role does MA have in transplantation services?*
- What is the authorization process like for in-home dialysis? Transplantation?
- We expect that as an MA plan, you're thinking of ways to help people with ESRD avoid hospitalizations and higher cost interventions. How are these efforts going? *Probes: What strategies have been most successful? What strategies haven't been as successful? What are the challenges?*

Benefit Design:

- How has expanding MA to people with ESRD affected your benefit design in general?
- How does your plan design benefits to help social needs such as poverty, transportation challenges, food insecurity, and others?
- What benefits are still needed? *Probes: How does your plan prioritize which benefits to offer?*
- What populations or communities might still need extra outreach (age, minorities, other)? *Probe: How could this outreach be done?*

Partnerships:

- What are your interactions with dialysis providers like? (for example, regarding payment incentives, contracting, and network design, other) *Probes: What goes well? What doesn't go so well? How could they be improved?*
- How do you use provider feedback?
- We understand that the dialysis market is dominated by some large players. Please tell us how your interactions differ based on size of the dialysis organization.

Perspectives:

- We're interested in understanding how expanding MA to people with ESRD has gone thus far. *Probes: What impact has it had on access to care for people with ESRD? How can access be further improved?*
- What about quality of care for people with ESRD? *Probe: What about outcomes?*
- What, if anything, have you heard from patients about out of pocket costs? *Probes: transportation? formularies?*
- How do you think expanding eligibility to people with ESRD will affect your plan? *Probe: How will it affect your beneficiaries with ESRD?*
- Is there anything else you feel is important for us to know about how your plan has responded to this policy change enabling ESRD patients to enroll in Medicare Advantage?

Thank you and incentive logistics

© 2025 Brazier JF et al. *JAMA Network Open*.

Kidney Care Management Company Interview Guide

Introductory and consent to audio record language

Background:

- What is your position and how long have you been in this role? *Probes: How did you first learn of the change in eligibility for individuals with end stage renal disease or ESRD?*

Preparation:

- Please describe your company mission and your care model.
- What makes your company distinct compared to other organizations that work with insurers to improve care for people with kidney disease?
- When was your company formed? *If after 2016*: How, if at all, did its formation relate to the 21st Century Cures Act?

Care Processes:

- How do you work with MA plans to improve care for people with kidney disease? *Probes: At what point in the chronic kidney disease trajectory are you trying to identify patients? Does your approach differ based on where patients are in their disease trajectory?*
- How might care be different for persons with MA compared to those with traditional fee-for-service Medicare? *Probes: How big a factor is transitioning people to palliative care? Or to home dialysis? Transplantation?*
- Do MA plans market their partnership with you to their potential members?
- What role do MA plans have in the choice of dialysis provider? *Probes: In deciding home versus in-center dialysis? Section of transplantation services?*

Partnerships:

- Please describe your approach to contracting with MA plans. *Probes: Do you use risk-based contracts? What metrics is your risk based on? Are you at risk for a plan's entire population of CKD patients or just those who agree to your service?*
- What have been your successes in contracting with MA plans? *Probes: Have you experienced growth? If so, why? What have been the challenges?*
- How have relationships with MA plans been initiated? *Probes: How are they maintained? (Ask about competition). Does your organization interact with MA plan staff on a regular basis? How so? Which types of staff? What are the benefits of this interaction for patients? For your organization? How burdensome is this, administratively?*
- How do you work with providers to improve care for people with chronic kidney disease? *Probes: How do you work with a patient's PCP and nephrologist? What roles are involved? (If have in-house clinicians, how do they work with patients' existing providers?)*
- How do you work with dialysis providers? *Probes: What goes well? What are your thoughts on the adequacy of MA plans' dialysis facility networks? How about network adequacy for other providers, like nephrologists?*
- How do you work with dialysis provider organizations to improve care for people with kidney failure? Are these relationships contractual? *(if yes: What are the metrics? how is risk handled? what are the challenges? successes?)*

Perspectives:

- How has Medicare's policy to open MA enrollment to persons with ESRD affected care for persons with kidney failure? *Probes: What about quality of care? What about access to care?*
- What about access and outcomes for people of lower SES, racial/ethnic minorities, sexual orientation, rural, other? *Probe: How can access and quality of care be further improved?*
- How has the policy impacted costs for persons with kidney failure?
- What more should MA plans do to improve care for people with kidney failure? *Probe: What about for people with chronic kidney disease more broadly?*
- Is there anything else you feel is important for us to know that we haven't talked about?

Thank you and incentive logistics

© 2025 Brazier JF et al. *JAMA Network Open*.

Dialysis Organization Leadership Interview Guide

Introductory and consent to audio record language

Background:

- What is your position here and how long have you been in this role?
- Please tell me how you first learned of the change in MA eligibility for individuals with ESRD.

Preparation:

- What did your organization do to prepare for the change?
- How have you addressed staff training? *Probes: hiring/developing new roles? engaging with MA to understand needs? engaging with patients? Anything else?*

Care Processes:

- How might care be different for persons with MA compared to those with traditional fee-for-service Medicare?
- What kinds of benefits do MA plans aim to provide? *Probe: How do those fit with your services?*
- What role does MA have in deciding home versus in-center dialysis? *Probe: What about in selecting transplantation services?*
- We've heard that for patients one of the largest barriers to getting on a transplant wait list is to have all dental work done ahead of time. What are your thoughts on this?
- What should MA plans do to improve care for people with ESRD?
- How has Medicare's policy to open MA enrollment to persons with ESRD affected the quality of care for persons with ESRD? *Probe: What about access to care?*
- What about access and outcomes for people of lower SES, racial/ethnic minorities, sexual orientation, rural, other? *Probe: How can access and quality of care be further improved?*
- How do you think outcomes of care will be affected overall?

Partnerships:

- How does your organization interact with MA plans in terms of contracting, authorizations, networks, or other important ways? *Probe: How burdensome is this, administratively?*

Perspectives:

- To what extent have patients who've switched to MA switched back? *Probe: What do you understand are some of their reasons for switching?*
- How might expanding MA eligibility to people with ESRD affect your organization financially? *Probe: What factors make it profitable?*
- How does this affect patients financially?
- How is your organization involved in helping patients navigate insurance and treatment decisions?
- In this conversation you've mentioned a few challenges and benefits from the new policy. What other benefits and challenges do you see?
- It's been approximately two years since this policy change went into effect - how has it met/not met your expectations?
- Anything else we haven't talked about?

Thank you and incentive logistics

© 2025 Brazier JF et al. *JAMA Network Open*.

Dialysis Organization Site Staff Interview Guide

Introductory and consent to audio record language

Background:

- What is your role at your facility? *Probe: How long have you been in this role?*
- I'm hoping you can tell me a little about the organization your facility is part of. *Probes: In your region how many facilities are there, what is the size, the profit status?*
- I'm hoping you can tell me a little about your patients. *Probes: About how many patients at your facility receive dialysis? In-center? In-home dialysis? The demographics of your patients? (age, ethnicity, socioeconomic status)*
- How would you describe the health of your patients? *Probe: comorbidities?*

Preparation:

- Please tell me how you first learned that ESRD patients can now enroll in Medicare Advantage plans. *Probe: What do you think of this expansion?*
- How did you prepare for this expansion of MA? *Probes: How have you addressed staff training? hiring/developing new roles? engaging with MA to understand needs engaging with patients? Anything else?*

Care Processes:

- What benefits do MA plans provide for people with ESRD in your facility?
- Do they provide care coordination and social work services? *Probe: How do these services coordinate with the services you provide?*
- What role does MA have in the selection of in-home versus in-center dialysis? *Probe: Transplantation services?*
- What other benefits or services should MA plans offer to people with ESRD?
- What are the steps to get services from the MA plan for your patients with ESRD? *Probes: How do you get authorizations for services? How burdensome is this process?*
- How are contracts between MA plans and your facility initiated?
- How do you maintain relationships with plans? *Probe: Do you have regular meetings?*

Partnerships:

- How do you interact with the dialysis provider organization? *Probes: on policies and procedures? staff training? oversight? what does your role consist of?*
- Providers sometimes do not know who has traditional Medicare and who has MA. How does knowing or not knowing the type of insurance coverage affect how providers treat patients with ESRD?
- Approximately how many patients in your facility have switched to MA since the expansion?

Perspectives:

- How might care differ for persons with MA versus those with traditional fee-for-service Medicare?
- What do you hear from ESRD patients about their experiences with MA? *Probes: reasons for enrolling in MA? satisfaction with care? reasons for disenrolling? unmet needs? barriers? Anything else?*
- What has been the financial impact of the expansion of eligibility for MA for your patients? *Probe: What about for dialysis facilities?*
- What are the negative consequences that could result from the expanded enrollment in MA? *Probe: What about positives?*
- Is there anything else we should know about the impact of this policy change?

Thank you and incentive logistics

© 2025 Brazier JF et al. *JAMA Network Open*.

eTable. Organization Characteristics and Participant Experience

**MA Plan***	**National/ Regional**	**Region**	**Size/Enrollment**	**Roles/Titles**	**Years in Role**
MA 1	National	National	1-5 million	Chief Medical Officers<br>VP/Directors Government Relations<br>VP/Directors of Care Management<br>VP/Directors of Clinical Operations	1y-5y
MA 2	Regional	Midwest	1-5 million		
MA 3	National	National	>5 million		
MA 4	Regional	West	<1 million		
MA 5	Regional	Southeast	<1 million		
MA 6	Regional	West	>5 million		
MA 7	Regional	Northeast	<1 million		
MA 8	Regional	Midwest	<1 million		
**KCM****	**National/ Regional**		**Size/Enrollment**	**Roles/Titles**	**Years in Role**
KCM 1	National	National: 50 states	>100,000	Regional/Chief Medical Officer<br>Chief Clinical Officer	<1y-5y
KCM 2	National	National: 50 states	>100,000		
KCM 3	Regional	National: <50 states	>100,000		
KCM 4	Regional	National: <50 states	>100,000		
KCM 5	Regional	National: <50 states	<100,000		
**DO-leadership*****	**National/ Regional**	**Region**	**Size**	**Roles/Titles**	**Years in Role**
DO 1	National	National	>100,000	Chief Medical Officers/Financial Officers<br>VP/Directors of Insurance Coordination<br>Directors of Communication<br>Facility owner/operator	3y-11y
DO 2	National	National	>100,000		
DO 3	Regional	Northeast	<100,000		
DO 4	Regional	West	<100,000		
DO 6	Regional	Southeast	<100,000		
DO 7	Regional	Midwest	<100,000		
**DO-site staff******	**National/ Regional**	**Region**	**Size**	**Roles/Titles**	**Years in Role**
SS1	National	National	>100,000	Social Workers<br>Insurance/Billing Coordinators/Specialists<br>Patient Accounts Managers<br>Nurse Care Managers	2y-23y
SS3	Regional	Northeast	<100,000		
SS4	Regional	West	<100,000		
SS6	Regional	Southeast	<100,000		
SS7	Regional	Midwest	<100,000		
SS8	Regional	Northeast	<100,000		

*MA=Medicare Advantage **KCM=Kidney Care Management ***DO=Dialysis Organization ****SS=Site Staff

© 2025 Brazier JF et al. *JAMA Network Open*.
