# Supplementary material for: Expansion and Marketing of Medicare Advantage to Persons With End-Stage Kidney Disease
Source: JAMA Netw Open. 2025 Jun 17;8(6):e2516359. doi: 10.1001/jamanetworkopen.2025.16359 (PMC12175025; doi:10.1001/jamanetworkopen.2025.16359)
Supplement: Supplement 2. — Data Sharing Statement [file jamanetwopen-e2516359-s002.pdf]

# Data Sharing Statement

Brazier. Expansion and Marketing of Medicare Advantage to Persons With End-Stage Renal Disease. *JAMA Netw Open*. Published June 17, 2025.
doi:10.1001/jamanetworkopen.2025.16359

**Data**
**Data available:** No
